# Human Factors: Do They Impact Surgical Performance?

**DOI:** 10.7759/cureus.69507

**Published:** 2024-09-16

**Authors:** Jerry Sam, Mahak Baid, Karthikeyan Dhandapani

**Affiliations:** 1 Trauma and Orthopaedics, Aneurin Bevan University Health Board, Newport, GBR

**Keywords:** human factors, music, patient safety, surgical performance, training

## Abstract

Introduction: Human factors in surgery relate to the environmental, organisational, and human factors that can impact performance in the operating theatre. This study assesses whether various factors such as music, counting backwards, and attempting to do simultaneous tasks impact surgical time and performance.

Methods: Ten orthopaedic surgical staff were asked to guide a metal loop around a metal maze in a ‘don’t buzz the wire’ game. The primary outcomes were the course completion time and the number of times the loop touched the wire. They were asked to do the course four times: one control run, with music, whilst counting backwards from a hundred in increments of three, and whilst simultaneously verbalising the steps of a dynamic hip screw (DHS) fixation.

Results: The average time to complete the course for the control was 33.8 seconds. This was similar to when music was played (33.4 seconds) but increased when counting backwards (38.7 seconds) and verbalising the steps of a DHS (69.8 seconds, p = 0.0039). The average number of touches for the control was 4.3. Similar findings were obtained when counting backwards (four touches), but the number of touches decreased when music was played (2.2 touches). The average number of touches increased to 10.6 when verbalising the steps of a DHS (p = 0.0078).

Conclusion: Human factors can affect surgical performance, and an awareness of this is vital to take necessary steps to minimise the impact this has on behaviour and performance in the operating theatre.

## Introduction

Human error is unavoidable and is part of human nature [1]. Two-thirds of all adverse events are surgical, and over half of these errors are preventable [2]. Much has been learnt about preventing error and adverse events from the aviation industry, and the environment in the cockpit and operating theatre have some similarities but also unique differences [3]. However, both settings require precision, communication, and teamwork. The operating theatre differs due to the unpredictability of human anatomy, variability of surgical procedures, and dynamics of surgical teams. Errors in the operating theatre can be caused by personal factors such as being tired or angry or system/organisational factors [3], which can be visually described by the Swiss cheese model [4]. This model postulates that failures can be active (errors caused by people at the human-system interface) and latent (errors committed as a result of inadequacies in the system or institution) [4]. When the ‘holes’ in the ‘cheese’ align, an error can bypass the various layers of defence, resulting in an adverse event [4]. Understanding these concepts is essential when formulating strategies to prevent adverse events in the operating theatre.

Despite various studies examining the impact of human factors on surgical performance, there remains a need to quantify the specific effects of everyday distractions and cognitive loads in a controlled environment. This study takes a unique approach, evaluating how various human factors, such as background music, mental distractions, and multitasking, affect surgical performance.

The key objectives of this study are to assess the effect of listening to music on task completion time and error rates during a simulated surgical task; to determine the impact of cognitive load introduced by counting backwards on surgical task performance; and to evaluate how multitasking affects surgical accuracy and completion time, such as verbalising surgical steps while performing a manual task.

## Materials and methods

This study was conducted in a trauma and orthopaedic unit at a district general hospital to investigate how different human factors impact surgical performance. The study utilised a controlled simulation-based approach to evaluate the effects of various distractions on fine motor skills and concentration, which are crucial in the operating theatre.

Participant selection

Ten orthopaedic surgical staff members were recruited for the study. The participants comprised eight orthopaedic registrars, one core surgical trainee, and one surgical care practitioner. Although there is a slight variance in experience and training levels, all participants were selected based on their active involvement in orthopaedic surgical procedures, which ensures a relevant comparison within the study's context. The inclusion and exclusion criteria were defined to provide a homogeneous sample pertinent to the study's aims.

Inclusion Criteria

Participants must be active members of the orthopaedic surgical staff and regularly involved in surgical procedures. Participants must have at least one year of surgical experience to ensure familiarity with operating room protocols, procedures, and basic surgical skills. After explaining the study's purpose, methodology, and expected involvement, consent must be obtained from each participant.

Exclusion Criteria

To maintain consistency in baseline motor skills, participants with any known neurological or motor impairments that could affect their performance in the task were excluded. Individuals who reported prior experience with the 'don't buzz the wire' game were excluded to prevent any bias due to familiarity with the task. Participants with hearing impairments were also excluded since one of the tasks involved listening to music, which required an accurate perception of auditory stimuli.

Task development and selection

The tasks were carefully developed to replicate different cognitive loads and distractions that a surgeon might experience in an operating room setting. The tasks aimed to simulate scenarios that require focused concentration, multitasking, and managing distractions. They are outlined in Table 1 and are explained in detail below.

**Table 1:** Outline of tasks performed.

Task	Description of task
1	Trial run with no distractions (control)
2	Completion of the task with music (the same song played for all participants)
3	Completion of the task whilst counting backwards from one hundred in increments of three
4	Completion of the task whilst simultaneously verbalising the steps of performing a dynamic hip screw fixation

Control Task

The 'don't buzz the wire' game served as the control task. Participants were required to guide a metal loop around a metal maze without touching the wire. This task was chosen for its simplicity and ability to measure fine motor skills without additional distractions. It provided a baseline measure of each participant's performance under optimal conditions.

Listening to Music

For the second task, participants performed the 'don't buzz the wire' game while listening to a pre-selected song. The chosen song was a classical instrumental piece with a moderate tempo (70-90 beats per minute) and no lyrics, played consistently with moderate volume for all participants. Although music preferences vary widely among surgeons, the choice of music was standardized for study purposes. This type of music was selected based on previous research suggesting that instrumental music might have calming effects, potentially enhancing concentration or, conversely, acting as a mild distraction [5].

Counting Backwards From 100 in Increments of Three

The third task involved guiding the loop through the maze while simultaneously counting backwards from 100 in increments of three. This task was designed to introduce a cognitive load, requiring participants to divide their attention between mental arithmetic and manual motor tasks. Such dual-task scenarios are common in surgical settings, where surgeons must manage manual precision and cognitive decision-making.

Simultaneously Verbalising the Steps of a Dynamic Hip Screw (DHS) Fixation

For the fourth task, participants were required to guide the loop around the maze while simultaneously verbalising the steps for performing a DHS fixation, a standard orthopaedic procedure. This task aimed to simulate a scenario where a surgeon must think through a complex surgical process while managing a manual task, thereby introducing a significant cognitive and motor load. As the primary focus was on measuring the maze completion time and the number of touches, the accuracy of the verbalised steps was not evaluated; they were merely used to increase the cognitive load.

Music details

For the music task, all participants listened to 'Pachelbel's Canon in D', a classical instrumental piece without lyrics, chosen for its moderate tempo and calming nature. The music was played at a consistent volume level (approximately 60 dB) to simulate background music commonly encountered in operating theatres. This choice was informed by literature suggesting moderate music can either improve focus by reducing stress or act as a distracting factor [5].

Task repetition and data collection

Each participant completed all four tasks three times to account for individual variability and to ensure robust data collection. The mean value of the three attempts was calculated for each condition to provide a reliable performance assessment. The outcomes measured for each task were (1) completion time (in seconds), i.e., the time to complete the metal maze without touching the wire; and (2) the number of touches, i.e., the number of times the metal loop touched the wire during each task.

Data were recorded by the principal investigator, who observed the participants throughout the task to ensure adherence to the protocol and to maintain consistency.

Study protocol and environment

The study was conducted individually with each participant to prevent observational learning or performance bias. Participants were not allowed to observe their peers during the tasks. All sessions took place in a quiet room free from interruptions, except for the music task, where the background music was played. The principal investigator provided consistent instructions to all participants and recorded all measurements to ensure reliability and reduce variability.

Measures to protect participants

Although formal Institutional Review Board (IRB) approval was not required, several measures were implemented to ensure participant safety, privacy, and well-being. Informed consent was obtained after explaining the study's purpose, procedures, and potential risks. Data were anonymised and securely stored, and access was restricted to the research team to maintain confidentiality. Participants were informed of their right to withdraw at any time without penalty. The tasks involved minimal risk and were conducted in a quiet, controlled environment to reduce discomfort. The study was presented as non-evaluative, focusing on performance factors rather than professional skills, and the principal investigator was present to provide support and ensure comfort throughout the study.

Statistical analysis

The average completion time and number of touches were calculated for each task condition. A Mann-Whitney U test was performed to determine the statistical significance of differences between the control and experimental conditions. A p-value of <0.05 was considered statistically significant.

## Results

Demographic details of the participants

The study included 10 orthopaedic surgical team participants, including eight orthopaedic registrars, one core surgical trainee, and one surgical care practitioner. The participants varied in terms of age, sex, qualifications, and years of surgical experience, as detailed in Table 2.

**Table 2:** Demographic details of the participants.

Participant number	Age (years)	Sex	Qualification	Years of surgical experience
1	32	M	Orthopaedic Registrar	5
2	29	F	Orthopaedic Registrar	3
3	35	M	Orthopaedic Registrar	7
4	28	F	Core Surgical Trainee	2
5	40	M	Orthopaedic Registrar	10
6	33	M	Orthopaedic Registrar	6
7	31	F	Orthopaedic Registrar	4
8	38	M	Orthopaedic Registrar	9
9	45	M	Surgical Care Practitioner	15
10	36	F	Orthopaedic Registrar	8

Performance results

The time to completion and number of metal touches for each participant are recorded in Table 3 and Table 4, respectively. The average time to complete the course and the average number of touches for each of the four tasks (control, listening to music, counting backwards, and verbalising the steps of DHS fixation) were calculated and presented in the format of mean ± standard deviation (SD).

**Table 3:** Time to task completion (seconds). The values represent the mean ± standard deviation (SD) for three trials performed by each participant. Statistical test used: Mann-Whitney U test. P-values: Control vs. counting backwards (p = 0.16); control vs. listening to music (p = 0.85); control vs. verbalising dynamic hip screw (DHS) steps (p = 0.0039).

Participant number	Control (mean ± SD)	Counting backwards (mean ± SD)	Listening to a song (mean ± SD)	Verbalising the steps of a DHS (mean ± SD)
1	49 ± 1.0	59 ± 2.0	39 ± 1.5	75 ± 3.5
2	30 ± 2.0	40 ± 1.0	29 ± 1.5	90 ± 4.0
3	19 ± 1.5	22 ± 0.5	18 ± 2.0	47 ± 2.5
4	20 ± 0.5	42 ± 3.0	22 ± 1.0	49 ± 2.0
5	38 ± 2.5	30 ± 2.0	28 ± 1.5	51 ± 3.0
6	30 ± 1.0	22 ± 0.5	28 ± 1.0	22 ± 2.0
7	23 ± 2.0	32 ± 1.5	30 ± 1.0	72 ± 3.5
8	32 ± 1.0	45 ± 3.0	40 ± 2.0	100 ± 5.0
9	55 ± 3.0	43 ± 2.0	48 ± 3.0	100 ± 6.0
10	42 ± 1.5	52 ± 2.5	52 ± 1.5	92 ± 4.5

**Table 4:** Number of metal touches. The values represent the mean ± standard deviation (SD) for three trials performed by each participant. Statistical test used: Mann-Whitney U test. P-values: Control vs. counting backwards (p = 0.74); control vs. listening to music (p = 0.054); control vs. verbalising dynamic hip screw (DHS) steps (p = 0.0078).

Participant number	Control (mean ± SD)	Counting backwards (mean ± SD)	Listening to a song (mean ± SD)	Verbalising the steps of a DHS (mean ± SD)
1	2 ± 1.0	3 ± 1.5	1 ± 0.5	15 ± 2.0
2	1 ± 0.5	6 ± 1.0	1 ± 0.5	9 ± 1.5
3	3 ± 1.0	3 ± 0.5	2 ± 1.0	6 ± 1.5
4	4 ± 0.5	1 ± 1.0	5 ± 0.5	7 ± 1.0
5	5 ± 1.5	1 ± 1.0	2 ± 0.5	25 ± 3.0
6	8 ± 2.0	6 ± 1.0	4 ± 1.0	19 ± 2.5
7	7 ± 1.0	4 ± 1.0	3 ± 0.5	4 ± 1.5
8	4 ± 0.5	7 ± 1.5	2 ± 0.5	3 ± 1.0
9	8 ± 1.5	4 ± 1.0	2 ± 1.0	26 ± 3.5
10	8 ± 1.0	6 ± 1.0	0 ± 0.5	12 ± 2.0

The performance results showed variations in time to complete the task and the number of errors across different conditions. In the control task, participants completed the course in an average of 33.8 ± 10.1 seconds with 4.3 ± 2.7 touches. When listening to music, the average time to complete the course was similar at 33.4 ± 11.5 seconds, but the number of touches decreased to 2.2 ± 2.0, suggesting a reduction in errors. However, when participants were asked to count backwards, the time increased to 38.7 ± 11.4 seconds, with an average of 4.0 ± 2.0 touches, indicating a slight increase in task duration but no significant change in errors. The most significant impact was observed when participants were required to simultaneously verbalise the steps of a DHS fixation; the average time to complete the course increased markedly to 69.8 ± 20.9 seconds, with a significant rise in the number of touches to 10.6 ± 7.1, highlighting the detrimental effect of high cognitive load on surgical performance.

## Discussion

The study of human factors predominantly relates to the individual and the system [6]. Although the two entities are intimately linked, they can also be two challenges that must be addressed separately [6]. Most of the literature has centred around studying and modifying the system or institution by focusing extensively on the operating room environment and team dynamic [6]. Our study predominantly focussed on the effects of external factors and stressors on surgical performance through simplified simulation scenarios. The results indicate a significant increase in time and errors when participants were asked to perform a high cognitive load task (verbalising the steps of DHS fixation), while listening to music had minimal impact on the completion time and even reduced the number of errors.

Environmental factors relating to human factors include operating room layout, the effect of noise, and team set-up. Congestion in the operating theatre can be caused by crowding of equipment, multiple lines, and a high volume of staff in the theatre [7]. This can create distractions and result in errors [7]. Various strategies have been proposed to overcome this by utilising all free space in the theatre to avoid overcrowding and having a colour-coded system for wires to prevent clustering [8]. In addition, noise is a well-documented factor that can result in lapses in concentration for the surgical team [9]. In our study, listening to music was found to have no difference in time to completion of the activity and decreased the number of errors (metal touches). This has been mirrored in other studies, which have demonstrated the positive effects of music on surgical performance by decreasing stress and anxiety, increasing situational awareness and creating a more relaxed environment [5]. However, the presence of music and noise should be differentiated. There are several sources of noise in the operating theatre, including alarms and monitors, the noise of people entering and exiting the operating room, and the sound of pagers [10]. This can negatively affect the surgeon's ability to concentrate by masking acoustic cues (related to the operation being performed) and impair communication between the surgical team [6]. Therefore, reducing noise in the operating room may positively impact surgical performance [11,12]. Various strategies have been proposed, including limiting the number of people in the theatre, restricting the use of pagers, and reducing the number of non-work related conversations whilst operating [13]. However, this may only sometimes be a practical approach, particularly in long cases and may create disagreements amongst the theatre team [13]. Therefore, a more balanced approach may be used to limit noise in the critical parts of the operation, a similar approach used in the aviation industry known as the 'sterile cockpit' [14]. This creates an environment free of unnecessary distractions and can help improve patient safety when applied appropriately in certain parts of the procedure [14].

While the environment and system can have several effects on human factors, personal issues relating to the individual surgeon can also play a crucial part and are an area of research that needs to be better developed [15]. In our study, we aimed to introduce various 'stressors' and distractions to observe if this would affect performance. The number of errors and time to completion of the activity increased by more than double when participants were asked to perform the activity and simultaneously verbalise the operative steps of a dynamic hip screw. Understanding this stress response and its relation to surgical errors is crucial. It should be considered in two dimensions: stress in the operative environment and personal stress and emotion. Moorthy et al. assessed the effect of multiple stressors, e.g., time pressure and noise, on surgical performance in theatre. They found that participants had impaired manual dexterity and increased errors [16]. The role of personal stress is an under-reported phenomenon in the medical community as it is often associated with stigma and fear of being viewed as 'weak' [17], and these learned behaviours can usually develop in a clinician's early years of training [18]. All factors in tandem can negatively affect a surgeon's technical and non-technical skills, such as communication and teamwork [15].

De Leval et al. [19] summarised succinctly the approach needed to address errors in the operating theatre with this statement: 'A marker of surgical excellence is not error-free performance but rather the ability to manage the mistakes and problems during an operation'. These skills can be taught in a simulated environment, allowing trainees to acquire the skills needed to deal with unexpected and stressful events in the operating theatre [20]. However, this training is not universal, and the question remains for the surgical training community: Is it time to introduce this teaching in the surgical curriculum to ensure all trainees are exposed to this necessary training?

Limitations

Not all participants had the same training level, so they would have differing technical abilities, which could influence the results. Only 10 participants were included in the study group. Hence, more extensive studies are needed to generalise the results to the broader community. Finally, the tasks performed by the participants could be more complex and provide a complete simulation of the operating room environment.

## Conclusions

Understanding human factors is essential to creating a safe operating room environment and enhancing patient safety. Interventions can be at the system or institution level or the personal level. These skills are essential attributes for the surgeon and must be developed in the early stages of training.
